# The Role of Aurora Kinase A in HBV-Associated Hepatocellular Carcinomas: A Molecular and Immunohistochemical Study

**DOI:** 10.3390/diagnostics16010160

**Published:** 2026-01-04

**Authors:** Mustafa Huz, Nese Karadag Soylu, Ahmet Koc, Zeynep Kucukakcali, Nefsun Danis, Onural Ozhan

**Affiliations:** 1 Department of Medical Pathology, Faculty of Medicine, Inonu University, Malatya 44050, Türkiye; 2 Department of Medical Genetics, Faculty of Medicine, Inonu University, Malatya 44050, Türkiye; 3 Biostatistics and Medical Informatics A.D., Faculty of Medicine, Inonu University, Malatya 44050, Türkiye; 4 Department of Medical Pharmacology, Faculty of Medicine, Inonu University, Malatya 44050, Türkiye

**Keywords:** HBV-related HCC, cryptogenic HCC, Aurora kinase A, phospho-PLK1, phospho-P53, BRCA1

## Abstract

**Objectives:** Although Aurora kinase A (AURKA) expression has been investigated in many cancer types, studies focusing on its role in hepatitis B virus-associated hepatocellular carcinoma (HBV-HCC) are limited. In this study, we examined the activity of AURKA and its substrates (PLK1, P53, and BRCA1) in HBV-HCC and cryptogenic hepatocellular carcinoma (Cr-HCC) cases. **Methods:** The study groups consisted of HBV-HCC, Cr-HCC, and healthy liver tissue cases. AURKA copy number variation (CNV) was analyzed using molecular methods. AURKA expression was evaluated by molecular and immunohistochemical (IHC) methods. AURKA substrates P53^Ser315^, PLK1^Thr210^, and BRCA1 were also analyzed by IHC. **Results:** There was no increase in AURKA gene copy number among the groups (2^−∆∆Ct^ < 2). AURKA level was significantly increased in both test groups (*p* < 0.001). At the protein level, AURKA was significantly higher in both cancer groups compared to the control group (*p* < 0.001). Phospho-P53^Ser315^ levels were significantly higher in both HBV-HCC and Cr-HCC groups compared to the control group (*p* = 0.002 and *p* < 0.001, respectively). Cr-HCC cases also showed significantly higher levels compared to HBV-HCC (*p* = 0.025). For phospho-PLK1^Thr210^, Cr-HCC cases showed statistically higher expression compared to both the control group and HBV-HCC cases (*p* = 0.001).

## 1. Introduction

Hepatocellular carcinoma (HCC) is the most common histological subtype of primary liver cancer and remains one of the leading causes of cancer-related mortality worldwide. Although substantial progress has been made in understanding the epidemiology and molecular mechanisms of HCC, targeted and personalized therapeutic strategies are still limited [1,2]. Protein kinases, including members of the Aurora kinase family, have emerged as critical mediators of oncogenesis and as important therapeutic targets in cancer drug development [3,4,5].

Recent advances in systemic therapy for HCC, particularly the introduction of immune checkpoint inhibitors and combinations such as atezolizumab plus bevacizumab, have demonstrated superior overall survival outcomes and reshaped first-line treatment standards [6,7,8]. Furthermore, approaches integrating locoregional therapies with systemic agents, including transarterial chemoembolization (TACE)-based strategies, are being investigated to optimize therapeutic benefit in selected patient populations [7,8,9,10].

Aurora kinases, members of the serine/threonine kinase family, play essential roles in regulating the cell division cycle. AURKA, which phosphorylates multiple kinases and phosphatases, influences diverse cellular processes including cell cycle progression, centrosome regulation, bipolar spindle formation, and chromosome segregation [11,12]. During mitosis, AURKA-mediated phosphorylation triggers mitotic entry, whereas substrate dephosphorylation marks mitotic exit. Key AURKA substrates include Polo-like kinase 1 (PLK1), p53, and breast cancer type 1 susceptibility protein (BRCA1) [13,14]. AURKA and PLK1, both highly expressed during the G2/M phase, act synergistically by promoting CDC2 activation through positive feedback regulation. Overexpression of AURKA enables cells to bypass the G2 DNA-damage checkpoint, resulting in centrosome amplification and genomic instability features commonly observed across multiple cancer types. Collectively, these findings support the hypothesis that AURKA, through overexpression or impaired inhibition, may function as an oncogene [11,12,15].

In HBV-related HCC, the hepatitis B viral protein HBx interacts with and binds to p53, thereby suppressing key tumor-suppressive mechanisms [12,16,17,18]. Overexpression of AURKA has been shown to destabilize p53 through Ser315 phosphorylation, leading to loss of function, centrosome amplification, chromosomal instability, and oncogenic transformation, whereas AURKA inhibition stabilizes p53 and induces G2/M cell cycle arrest [17,19].

PLK1, a major substrate of AURKA, is a critical mitotic kinase that regulates several key steps in cell division [19,20,21]. Numerous studies have shown that PLK1 phosphorylation is essential for mitotic entry. AURKA directly phosphorylates the Thr210 residue within the PLK1 kinase domain, initiating mitotic progression [21,22,23,24]. A DNA-damage response is initiated at the centrosome following genomic insult, mediated by BRCA1, which coordinates DNA repair in the nucleus and prevents centrosome overduplication. Loss or mutation of BRCA1 has been associated with centrosome amplification and mitotic spindle abnormalities in cancer cells. BRCA1 has been reported to regulate centrosome homeostasis by binding to and phosphorylating AURKA [25].

In this study, we included HCC cases associated with HBV etiology as well as cryptogenic HCC (Cr-HCC) cases, which lack a clinically or serologically identifiable cause. This design enabled a more comprehensive interpretation of the findings. We investigated AURKA kinase activity on key substrates, including p53, PLK1, and BRCA1, alongside AURKA expression levels and gene copy-number alterations.

## 2. Materials and Methods

### 2.1. Creation of Study Protocol and Selection of Cases

The experimental groups were formed as follows: The first group (control group) consisted of liver samples from healthy living donors undergoing living-donor liver transplantation, whose livers were confirmed as histopathologically normal. The second group (HBV-HCC) included cases diagnosed with HBV-related hepatocellular carcinoma (HBV-HCC). The third group (Cr-HCC) comprised cases of cryptogenic hepatocellular carcinoma (Cr-HCC), which were serologically and clinically unrelated to any known etiology (Table 1).

Based on a power analysis, the total sample size was determined as be 51 cases, with a minimum of 17 cases per group. Cases were randomly selected retrospectively from archival records from 2021, with no restrictions on age or gender. For HBV-HCC cases with immunohistochemical (IHC) staining results showing HBV positivity (>30%) were preferred.

Hematoxylin-eosin-stained slides from all cases were re-evaluated. Tumor representative areas were selected for the preparation of paraffin blocks using the tissue microarray (TMA) technique. TMA paraffin block tissues, 3 mm in diameter, were utilized for molecular testing and IHC analysis [26]. The TMA blocks were prepared using a Harris Uni-Core Size: 3.0 punch (Electron Microscopy Sciences, Cat. 69036-30).

The expression level and copy number variation (CNV) of the AURKA gene were analyzed using molecular techniques. Downstream substrates of AURKA, including P53, PLK1, and BRCA-1, were examined using immunohistochemistry (IHC). Phospho-specific antibodies targeting AURKA-activated molecules, such as Phospho-PLK1^Thr210^ and Phospho-p53^Ser315^, were selected for analysis [17,27]. Given the challenges of performing gene expression analysis on formalin-fixed paraffin-embedded (FFPE) tissues, IHC-based analysis of AURKA was established as a contingency plan (Plan B) [28].

### 2.2. Molecular Genetic Analyzes

AURKA gene expression and copy number variation (CNV) were analyzed using SYBR Green-based quantitative PCR. The human single-copy gene 36B4 was used as the reference gene, and specific primers were designed for both gene expression and CNV analysis (Table 2).

**Figure 1:**
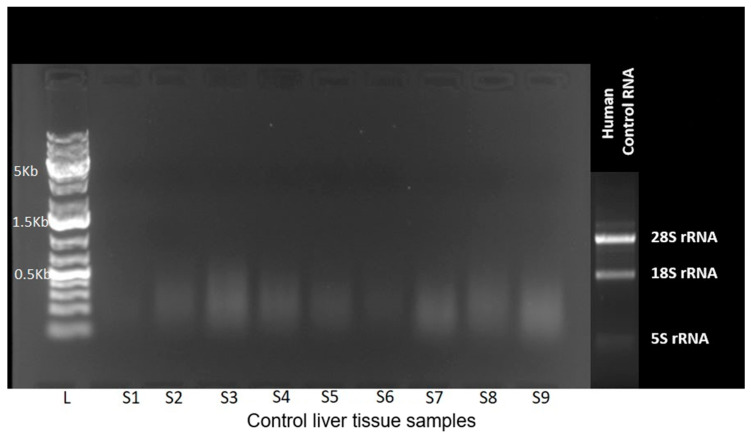
Agarose gel electrophoresis of RNA isolated from control liver tissue samples.

For gene expression analysis, 8 µm sections taken from TMA blocks of formalin-fixed paraffin-embedded (FFPE) liver tissues were transferred into 1.5 mL tubes, and RNA extraction was performed using the Monarch Total RNA Miniprep Kit (Cat. No: NEB #T2010, New England Biolabs, Ipswich, MA, USA). Sections were processed through xylene-based deparaffinization, ethanol washes, enzymatic digestion, and column-based RNA purification according to the manufacturer’s protocol. RNA quality was assessed via gel electrophoresis (1% gel, 80 V for 50 min), which revealed no intact band formation under UV light, indicating RNA fragmentation (Figure 1). Despite this, cDNA synthesis proceeded using the ProtoScript First Strand cDNA Synthesis Kit (Cat. No: E6560S, New England Biolabs, Ipswich, MA, USA).

For CNV analysis, 8 µm sections were obtained from FFPE tissue blocks, deparaffinized, and processed using the Quick-DNA FFPE Miniprep Kit (Cat. No: D3067, Zymo Research, Irvine, CA, USA). DNA quality control was performed using a NanoDrop spectrophotometer (ND-1000, Thermo Scientific, Waltham, MA, USA), and samples with an A260/A280 ratio of 1.8–2.0 were included in the analysis. A total of 200 ng DNA was used per reaction, with 10 µL Master Mix, 0.5 µL primer, 0.1 µL SYBR Green, and nuclease-free water added to each well. Real-time PCR was performed in triplicate using the Gotaq qPCR Master Mix Kit (Cat. No: A6001, Promega, Madison, WI, USA) for 40 cycles.

CNV analysis was conducted on the StepOnePlus Real-Time PCR System (Applied Biosystems, Waltham, MA, USA), and results were evaluated using the relative quantification method.

### 2.3. Immunohistochemical Analysis

Tissue microarray (TMA) paraffin blocks were sectioned at 4 µm using a microtome (RM2255, Leica Biosystems, Nussloch, Germany). Antigen retrieval and subsequent steps were performed on an automated IHC platform (Dako Omnis, Agilent, Santa Clara, CA, USA) following standard protocols. Primary antibodies included AURKA (1:100, GeneTex, Hsinchu City, Taiwan), Phospho-p53^Ser315^ (1:100, GeneTex, Taiwan), and BRCA1 (1:200, Elabscience, Houston, TX, USA), incubated for 30 min. For Phospho-PLK1^Thr210^ (1:100, Affinity Biosciences, Changzhou, China), low-pH antigen retrieval was applied, followed by a 60 min incubation. Detection was performed using the EnVision FLEX system (Agilent Dako, Santa Clara, CA, USA). DAB chromogen was applied, and slides were counterstained with hematoxylin for 10 min.

Stained slides were evaluated in two dimensions under a light microscope (Olympus BX53). Staining intensity was defined by color depth and assigned a semi-quantitative score (0–4). Staining prevalence (positivity rate) was defined as the percentage of positive TMA tissue area and assigned a semi-quantitative score (0–3). The scores from the two parameters were multiplied to obtain a final score ranging from 0 to 12 (Table 3) [29].

Two independent pathologists evaluated the IHC results. In cases where scoring differed, slides were re-examined jointly and a consensus score was reached.

### 2.4. Statistical Analysis

The Shapiro–Wilk test was used to assess the normality of the data. For numerical values that did not follow a normal distribution, the Mann–Whitney U test was applied for statistical analysis. Data were summarized as median (minimum-maximum). A *p*-value of <0.05 was considered statistically significant. All analyses were performed using IBM SPSS Statistics version 26.0.

## 3. Results

### 3.1. AURKA Gene Expression

In the AURKA gene expression study, spectrophotometric measurement and agarose gel electrophoresis following RNA isolation indicated RNA fragmentation (Figure 1).

RNA extracted from FFPE liver tissues showed no distinct 28S and 18S rRNA bands, indicating RNA degradation and unsuitability for gene expression analysis. The small panel shows a reference RNA sample run on a separate gel to illustrate the intact 28S and 18S bands expected in high-quality RNA.

The gel electrophoresis image was obtained from normal liver tissue samples (Control group). Despite fragmented RNA, cDNA synthesis and RT-PCR were performed as planned; however, no cDNA amplification was observed and no Ct values were generated, consistent with the RNA quantification and gel electrophoresis findings. These results confirm the known challenges of performing gene expression analysis on FFPE tissue samples, particularly those derived from hepatectomy specimens. Accordingly, AURKA expression was evaluated at the protein level by IHC, in line with the predefined contingency strategy (Plan B).

### 3.2. AURKA Gene Copy Number Variation

When HBV-HCC and Cr-HCC groups were compared with the control group, the 2^−ΔΔCt^ values were found to be <2 in both groups, indicating no increase in AURKA copy number relative to normal liver tissue. When the HBV-HCC and Cr-HCC groups were compared to each other after undergoing identical pre-analytical and analytical processing the 2^−ΔΔCt^ value was 2.11. However, this difference was not statistically significant (Table 4), confirming that no measurable copy number variation was present between the two HCC subtypes.

### 3.3. Immunohistochemical Markers Findings

Tissue sections from TMA paraffin blocks were stained using immunohistochemistry (IHC) to evaluate protein expression of AURKA, P53^Ser315^, PLK1^Thr210^, and BRCA1. Staining extent and intensity were assessed by light microscopy.

**Figure 2:**
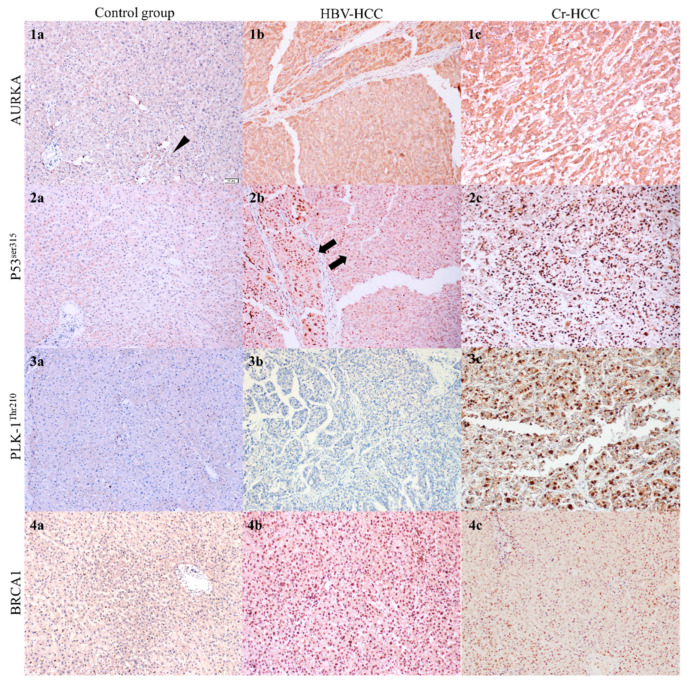
Immunohistochemical evaluation across study groups. (**1a**) Control liver tissue demonstrating no AURKA expression. In some cases, weak cytoplasmic staining was observed in pericentral hepatocytes (arrowhead), consistent with localized cellular stress in this region. Arrows indicate areas with focal cytoplasmic positivity. AURKA, ×100. (**1b**,**c**) HBV-HCC and Cr-HCC tissues showing moderate-to-strong AURKA positivity with predominant nuclear staining and variable cytoplasmic staining. Cytoplasmic staining is consistent with AURKA involvement in microtubule dynamics during cellular stress and mitotic regulation. AURKA, ×100. (**2a**) Control tissue negative for p53^Ser315^, ×100. (**2b**) HBV-HCC demonstrating moderate and diffuse nuclear p53^Ser315^ positivity; intratumoral staining heterogeneity observed (arrows), ×100. (**2c**) Cr-HCC showing strong and diffuse nuclear p53^Ser315^ staining, ×100. (**3a**,**b**) Control and HBV-HCC tissues negative for PLK1^Thr210^, ×100. (**3c**) Cr-HCC demonstrating strong and diffuse nuclear PLK1^Thr210^ staining, ×100. (**4a**) Control tissue negative for BRCA1, ×100. (**4b**) HBV-HCC with prominent nuclear BRCA1 staining, ×100. (**4c**) Cr-HCC with weak and focal nuclear BRCA1 staining, ×100.

AURKA immunostaining showed nuclear localization, consistent with its known role in centrosome and mitotic spindle regulation, but cytoplasmic staining was observed, particularly in conditions suggestive of microtubule stress or repair [30,31]. In the Control group, weak nuclear and cytoplasmic staining was detected in pericentral hepatocytes in a subset of cases likely reflecting early cytotoxic and enzymatic exposure in this zone while the remaining samples showed no staining. HBV-HCC cases exhibited diffuse nuclear staining accompanied by cytoplasmic positivity, whereas Cr-HCC cases demonstrated stronger, more widespread, and more heterogeneous staining patterns (Figure 2).

P53^Ser315^ staining was evaluated in the nucleus, cytoplasm, and mitochondrial matrix. In the Control group, five cases showed sparse and weak nuclear staining, while no staining was observed in eleven cases. HBV-HCC cases demonstrated predominantly moderate to weak staining, with one case showing two distinct staining intensities suggestive of tumor heterogeneity. Cr-HCC cases displayed strong nuclear staining in four samples, while the remaining cases exhibited moderate to weak staining (Figure 2).

PLK1^Thr210^ staining localized primarily to centrosomes, centromeres, and kinetochores in the nucleus, as well as to cytoskeletal structures in the cytoplasm. In the Control group, weak to moderate pericentral hepatocyte staining was observed in four cases. Eight HBV-HCC cases demonstrated no staining, and the remaining cases showed weak staining. In contrast, Cr-HCC samples generally exhibited moderate to strong staining, with four cases demonstrating intense diffuse positivity (Figure 2).

The results were summarized through statistical analysis and are presented in Table 5.

## 4. Discussion

In HCC developing on a cirrhotic background, diagnosis is often based on characteristic dynamic contrast-enhanced CT/MRI features and is standardized through the Liver Imaging Reporting and Data System (LI-RADS) framework. In at-risk cirrhotic patients, lesions meeting major imaging criteria and categorized as LI-RADS 5 are considered diagnostic of HCC, and biopsy is usually not required. This strategy is clinically justified given biopsy-related bleeding and needle-tract tumor seeding risks, as well as the possibility of false-negative results due to sampling limitations. However, biopsy remains relevant in non-cirrhotic patients, in indeterminate LI-RADS 3–4 observations, or when histologic, molecular, or immunophenotypic characterization is needed to guide management; the principal indications are summarized in Table 6 [32,33,34,35].

Beyond radiological diagnostic criteria, genomic alterations provide an additional biological framework for understanding hepatocellular carcinoma. In contrast to point mutations, copy number variations (CNVs) represent larger-scale genomic alterations that can influence gene dosage and expression levels. The impact of CNVs on oncogene amplification and transcriptional activity has been demonstrated across multiple cancer types [36,37]. For instance, hepatocellular carcinoma (HCC) frequently exhibits 2 to 10 fold amplification of oncogenes such as Cyclin D1, CDKN1A, KRAS, and MDM2 [32,38]. Although AURKA amplification has been reported in colorectal and breast cancers, prior work detected CNV in only three of 224 hepatitis B virus-negative HCC cases [37,38,39].

In our study, when comparing HBV-HCC and Cr-HCC cases to the Control group, no increase in AURKA copy number was observed (2^−ΔΔCt^ < 2). Comparisons between HBV-HCC and Cr-HCC groups, both derived from hepatectomy specimens processed under similar pre-analytical conditions, yielded a 2^−ΔΔCt^ value of 2.11, which was not statistically significant (*p* = 0.060). Mean Ct values for the reference gene were comparable in HBV-HCC and Cr-HCC (21.99 and 21.56, respectively), whereas the Control group demonstrated an earlier Ct value (20.98). This difference likely reflects the use of hepatectomy-derived tissue microarray samples in the two HCC groups. These findings are consistent with previous reports showing no significant AURKA CNV in HCC. However, inherent limitations, including sample variability and pre-analytical factors such as cold ischemia time, tissue size, and processing conditions, may have influenced the results [32,38].

Overexpression of AURKA has been reported particularly in colorectal adenocarcinoma, non-small cell lung cancer, and ovarian cancer, and has been associated with poor prognosis and treatment resistance [40]. Recently, the AURKA inhibitor MLN8237 (Alisertib) entered clinical trials, and experimental studies have demonstrated that MLN8237 exhibits a strong synergistic effect with Sorafenib in HCC models [41,42]. AURKA expression was analyzed on a protein level using immunohistochemistry (IHC) since RNA of adequate quality could not be obtained from the TMA tissue series. The inability to obtain high-quality RNA was correlated with the duration of cold ischemia in cases and was thus discussed under the “Study Limiting Factors” section.

Summarizing the IHC staining results, the AURKA-specific antibody demonstrated moderate to strong staining in the nuclei and cytoplasm of HBV-HCC and Cr-HCC cases, whereas half of the control samples showed no staining and the remaining exhibited only weak cytoplasmic staining. AURKA’s major function is related to centrosome regulation and spindle assembly during mitosis; however, cytoplasmic staining has been reported under cellular stress conditions due to its auxiliary role in microtubule organization [30,31].

When comparing HBV-HCC and Cr-HCC cases with the control group, AURKA immunoreactivity (including both nuclear and cytoplasmic staining patterns) was significantly higher in both tumor groups (*p* < 0.001). No significant difference was observed between HBV-HCC and Cr-HCC (*p* = 0.224). This staining pattern aligns with previous studies demonstrating increased AURKA activity in malignant tissues compared with non-tumoral liver tissue [43,44,45].

AURKA regulates the function of its substrates including tumor suppressor genes and oncogenes involved in mitosis through AURKA-mediated phosphorylation [17]. Three of these substrates (P53, PLK1, and BRCA-1) were analyzed in this study. The activity and functional relevance of AURKA on these molecules were evaluated across HBV-HCC, Cr-HCC, and Control groups.

AURKA has been shown to phosphorylate P53 at Ser315, leading to its ubiquitination by MDM2 and subsequent proteolysis. Under physiological conditions, P53 is not degraded in the presence of MDM2 if AURKA activity is low or absent. However, when MDM2 is mutated or AURKA is overexpressed as demonstrated in our study phosphorylation of P53 at Ser315 destabilizes P53 and disrupts checkpoint-mediated responses, thereby promoting oncogenic transformation [17,19,46,47].

In the Control group, phospho-P53^Ser315^ staining was absent in most cases, with only sporadic weak nuclear staining observed. In contrast, HBV-HCC and Cr-HCC cases exhibited marked P53^Ser315^ positivity, and both demonstrated statistically significant differences compared with the Control group (*p* = 0.002 and *p* < 0.001, respectively). Cr-HCC cases also differed significantly from HBV-HCC (*p* = 0.025). Although phosphorylated P53^Ser315^ was elevated in both HCC groups, staining intensity was greater in Cr-HCC. This finding suggests that P53 destabilization via AURKA-mediated phosphorylation, and its interaction with the P53-MDM2 axis, may differ mechanistically between HBV-HCC and Cr-HCC. These results imply that therapeutic approaches targeting AURKA overexpression may yield distinct clinical benefits depending on the underlying etiology, potentially offering greater therapeutic value in Cr-HCC.

PLK1, another key substrate of AURKA, is typically overexpressed in tumor cells. AURKA directly phosphorylates PLK1 at the conserved Thr210 residue within its activation loop, a modification essential for PLK1 activation. Without Thr210 phosphorylation, PLK1 remains inactive despite its association with target proteins [20,48,49,50]. Balanced PLK1 activity is required for proper cell proliferation and mitotic progression, and dysregulation particularly aberrant down-regulation has been associated with carcinogenesis [51]. Activation of PLK1 is necessary for progression through the G2/M checkpoint, and AURKA-mediated phosphorylation of Thr210 has been shown to promote mitotic entry [22,24,49,50,52].

In this study, a phospho-specific PLK1^Thr210^ antibody was used to assess the relationship between AURKA and PLK1. Cr-HCC cases demonstrated pronounced nuclear PLK1^Thr210^ staining, whereas no staining was observed in the Control group except for weak pericentral cytoplasmic staining in a few hepatocytes. This pericentral pattern is consistent with early liver injury due to zone 3 exposure to metabolic stress and toxic substances [53]. Statistically, PLK1^Thr210^ expression was significantly higher in Cr-HCC than in the Control group (*p* < 0.001) and HBV-HCC (*p* = 0.001). However, no significant difference was observed between HBV-HCC and Control groups (*p* = 0.296). These findings indicate that PLK1 activation differs by etiology and may be more relevant in Cr-HCC pathogenesis.

Overexpressed PLK1 undergoes degradation via the endonuclease-G pathway, leading to apoptosis, highlighting its relevance as a therapeutic target [49,50]. In our study, PLK1 phosphorylated at Thr210 an activation event mediated by AURKA was observed exclusively in Cr-HCC cases, whereas HBV-HCC tissues showed minimal phosphorylated PLK1. This divergence between p53^Ser315^ upregulation in HBV-HCC and PLK1^Thr210^ activation in Cr-HCC suggests distinct oncogenic programs driving the two subtypes.

Given that PLK1 inhibitors require phosphorylated, active PLK1 to exert their therapeutic effect, the absence of PLK1^Thr210^ in HBV-HCC may limit the utility of PLK1-directed therapies in this subgroup [24,25,52]. In contrast, the presence of activated PLK1 in Cr-HCC indicates a potentially actionable therapeutic axis in this etiology. However, functional evidence directly confirming the biological consequences of PLK1^Thr210^ activation in Cr-HCC such as its role in proliferation, tumorigenesis, or therapy response is currently lacking. Therefore, while our data suggest that targeting active PLK1 may hold therapeutic promise specifically in Cr-HCC, further mechanistic studies are required to validate and translate this approach [24].

In the evaluation of BRCA1 staining, HBV-HCC cases demonstrated significantly higher expression compared to the Control group (*p* = 0.006). However, no significant difference was observed between Cr-HCC and the Control group (*p* = 0.239), nor between the two tumor groups (HBV-HCC vs. Cr-HCC; *p* = 0.110). AURKA, localized to the centrosome, facilitates the G2/M transition through its kinase activity. Importantly, inactivation of BRCA1 also a centrosomal protein has been associated with loss of the G2/M checkpoint and increased AURKA phosphorylation [54].

Although both HCC groups showed similarly elevated AURKA expression, the BRCA1 results suggest a distinct regulatory pattern in HBV-HCC. In HBV-HCC, high AURKA expression appears sufficient to drive mitotic progression despite BRCA1 presence, supporting prior reports that AURKA overexpression can override BRCA1 mediated genomic surveillance and promote cell cycle progression even under DNA damage conditions [25,45,46].

Our findings suggest that AURKA may represent a potential therapeutic target, particularly in HBV-HCC and Cr-HCC, tumor subtypes characterized by genomic instability and dysregulated mitotic control. While AURKA and its substrate, PLK1, inhibitors have been extensively investigated in preclinical models and early-phase clinical studies in other malignancies, accumulating evidence indicates that their application in hepatocellular carcinoma has so far remained largely limited to preclinical and early translational settings, rather than routine clinical integration [15,17,20,42,55,56,57].

In Cr-HCC, increased AURKA expression accompanied by phosphorylation of PLK1 at Thr210 supports a disease mechanism predominantly driven by aberrant mitotic control [20,24]. In contrast, HBV-associated tumors demonstrated more pronounced alterations in p53 phosphorylation (via Ser315) and BRCA1-related pathways, consistent with disruption of DNA damage checkpoint regulation [16,20,24,46,49,52,56]. From a clinical perspective, these findings suggest that AURKA-targeted strategies may require etiological stratification, with PLK1-centered approaches potentially being more relevant in Cr-HCC, whereas therapeutic strategies focusing on the p53/BRCA1 axis may be more applicable in HBV-associated disease.

In the next five years, we anticipate increased research on the development of biomarker-guided treatment strategies targeting AURKA and PLK1 related pathways, driven by advances in precision oncology approaches, a better understanding of immune-metabolic signaling pathways, and high-resolution profiling of tumor biology. We believe this approach could complement emerging systemic treatment modalities and contribute to improved clinical outcomes in patients with advanced HCC.

### Factors Limiting the Study

Formalin-fixed paraffin-embedded (FFPE) liver tissue offers valuable archival material for molecular studies; however, sample processing conditions can significantly affect nucleic acid integrity. Consistent with previous reports, prolonged formalin exposure, large tissue volume, and intraoperative transfer delays likely contributed to the RNA fragmentation observed in our samples (Figure 1). Additionally, factors such as storage duration, processing variability, and cold ischemia in donor liver specimens may have further impaired RNA quality, preventing reliable transcript-level analysis even in control samples [53]. For this reason, molecular evaluation in our study relied primarily on immunohistochemistry and phospho-protein assessment rather than RNA-based assays.

The study also has biological and methodological limitations. Our findings suggest differences in AURKA-dependent downstream signaling pathways between Cr-HCC and HBV-HCC; however, these observations are based on a single-center cohort and require confirmation in larger, multicenter series. Additionally, while the clinical context of the cases was similar, variables such as age, sex, tumor stage, and liver function status were not analytically controlled, highlighting the need for validation in larger and clinically stratified cohorts. Furthermore, although PLK1 phosphorylation was detected in Cr-HCC and p53/BRCA1 modulation in HBV-HCC, functional studies involving AURKA inhibition and PLK1 modulation are needed to demonstrate whether AURKA directly mediates these effects. Finally, our study does not directly correlate with clinical outcomes; therefore, further studies evaluating mechanistic analyses in conjunction with clinical data will be necessary to determine the translational relevance of the observed pathway differences.

## 5. Conclusions

AURKA expression was found to be elevated in both HBV-HCC and Cr-HCC cases, consistent with reports in various other cancer types. However, the activity of AURKA on its downstream substrates (P53^Ser315^, PLK1^Thr210^, and BRCA1) differed between the two tumor groups. These findings suggest that AURKA-associated signaling pathways may operate through distinct molecular mechanisms in the initiation and progression of carcinogenesis in HBV-HCC and Cr-HCC.

AURKA-mediated phosphorylation of PLK1^Thr210^ was observed at a higher level in Cr-HCC cases than in HBV-HCC cases. Given the limited use of cytotoxic agents in HCC, antisense oligonucleotide mediated PLK1 inhibition has been proposed as a promising therapeutic strategy [19,49]. Based on our findings, targeting the active, phosphorylated form of PLK1 in Cr-HCC may provide greater therapeutic benefit than in HBV-HCC, where PLK1^Thr210^ activation was minimal. The intense nuclear staining pattern observed with the PLK1^Thr210^ phospho-specific antibody in Cr-HCC suggests that PLK1^Thr210^ may serve as a potential biomarker for this subgroup. Additionally, no alterations in AURKA gene copy number were detected in either HBV-HCC or Cr-HCC cases.

Our findings should be supported by future studies to further clarify the role and mechanisms of AURKA in HCC. A key limitation of this study is that all samples were obtained from a single institution, which may introduce bias related to demographic and regional characteristics and limit the generalizability of the results. Therefore, multicenter studies with larger and more diverse cohorts are needed to validate and extend these findings.

## Figures and Tables

**Table 1:** Study groups.

	Groups	Histopathologic Feature
Control	Normal liver tissue. Healthy donor liver sample.	Liver wedge biopsy material. TMA generated from paraffin block.
HBV-HCC	HBV-associated HCC cases	Hepatectomy material. TMA paraffin block, generating new tissue microarrays from paraffin block.
Cr-HCC	HCC cases that cannot be clinically and serologically associated with any etiology.	Hepatectomy material. TMA paraffin block generating new tissue microarrays from paraffin block.

**Table 2:** Primer sequence (from 3′ to 5′).

Primer	Primer Sequence
AURKA-F	GCAACCAGTGTACCTCATCCTG
AURKA-R	AAGTCTTCCAAAGCCCACTGCC
36B4-F	CAGCAAGTGGGAAGGTGTAATCC
36B4-R	CCCATTCTATCATCAACGGGTACAA

**Table 3:** IHC scoring criteria.

Staining Prevalence	Score	Color Depth	Score
Staining rate of cells 0%	0	No staining	0
Staining rate of cells 1–25%	1	Weak	1
Staining rate of cells 25–50%	2	Middle	2
Staining rate of cells 50–75%	3	Strong	3
Staining rate of cells 75–100%	4	—	—

**Table 4:** CNV statistical results compared between HBV-HCC and Cr-HCC.

Variables	HBV-HCC	Cr-HCC	*p* Value
	Median (Minimum–Maximum)		
∆∆Ct	6.981 (4.454–9.983)	6.181 (3.753–8.11)	0.060
2^−∆∆Ct^	0.1 (0.012–0.577)	0.174 (0.046–0.938)	0.060

**Table 5:** IHC parameters and statistical analysis.

	Median (Minimum–Maximum)		
Variables	Control Group	HBV-HCC	*p* Value
**AURKA**	0 (0–1)	9 (4–12)	<0.001 *
**P53Ser315**	1 (0–2)	2 (0–6)	**0.002 ^¥^**
**PLK1Thr210**	1 (0–6)	2 (0–8)	0.296
**BRCA1**	1 (0–2)	4 (0–8)	**0.006 ^₫^**
	**Control group**	**Cr-HCC**	***p***
**AURKA**	0 (0–1)	8 (3–12)	<0.001 *
**P53Ser315**	1 (0–2)	4 (0–9)	**<0.001 ^¥^**
**PLK1Thr210**	1 (0–6)	6 (0–12)	**<0.001 ^Ꝋ^**
**BRCA1**	1 (0–2)	1 (0–4)	0.239
	**HBV-HCC**	**Cr-HCC**	***p***
**AURKA**	9 (4–12)	8 (3–12)	0.224
**P53Ser315**	2 (0–6)	4 (0–9)	**0.025 ^¥¥^**
**PLK1Thr210**	2 (0–8)	6 (0–12)	**0.001 ^Ꝋ^**
**BRCA1**	4 (0–8)	1 (0–4)	0.11

Bold values indicate statistically significant differences between the compared groups. *; Control vs. HBV-HCC and Control vs. Cr-HCC, ¥; Control vs. HBV-HCC, ¥¥; HBV-HCC vs. Cr-HCC, Ꝋ; Cr-HCC vs. Control and Cr-HCC vs. HBV-HCC, ₫ **;** Control vs. HBV-HCC. A statistically significant increase in AURKA was observed in HBV-HCC and Cr-HCC groups compared to the Control group (*p* < 0.001 *). There was no significant difference in the high level expression of AURKA between both experimental groups (*p* = 0.224). Phosphospecific P53^Ser315^ was elevated in HBV-HCC and Cr-HCC groups and was statistically significant compared to the Control group (*p* = 0.002 ^¥^, *p* < 0.001 ^¥^, respectively). However, when HBV-HCC and Cr-HCC groups were compared, the high expression of p53^Ser315^ in HBV-HCC group differed from Cr-HCC group (*p* = 0.025 ^¥¥^). Phosphospecific PLK1^Thr210^ was highly expressed in the Cr-HCC group, differing from the Control group (*p* < 0.001 ^Ꝋ^) and the HBV-HCC group (*p* = 0.001 ^Ꝋ^) and this difference was statistically significant. BRCA1 was found to be expressed significantly higher in the HBV-HCC group as a result of statistical analysis (*p* = 0.006 ^₫^).

**Table 6:** Clinical Role of Imaging and Liver Biopsy in the Diagnosis of Hepatocellular Carcinoma.

Clinical Scenario	Role of Imaging (LI-RADS)	Indication for Liver Biopsy
Cirrhotic patient with LI-RADS 5 lesion	Diagnostic	Not required
Cirrhotic patient with LI-RADS 3–4 lesion	Indeterminate	May be required for diagnostic confirmation
Non-cirrhotic patient	Limited diagnostic specificity	Generally required
Atypical imaging features	Insufficient for definitive diagnosis	Required for differential diagnosis
Patients considered for systemic chemotherapy	Limited role in treatment selection	May be required for molecular subclassification or target identification
Patients considered for immunotherapy	Not diagnostic	May be required for immunophenotypic profiling (e.g., PD-L1, TME)
Assessment of treatment response or disease progression	Primary follow-up tool	Selected cases only (suspected resistance or transformation)

## Data Availability

The original contributions presented in this study are included in the article. Further inquiries can be directed to the corresponding author.
